# Effect of Eugenol on Cell Surface Hydrophobicity, Adhesion, and Biofilm of *Candida tropicalis* and *Candida dubliniensis* Isolated from Oral Cavity of HIV-Infected Patients

**DOI:** 10.1155/2014/505204

**Published:** 2014-04-03

**Authors:** Suelen Balero de Paula, Thais Fernanda Bartelli, Vanessa Di Raimo, Jussevania Pereira Santos, Alexandre Tadachi Morey, Marina Andrea Bosini, Celso Vataru Nakamura, Lucy Megumi Yamauchi, Sueli Fumie Yamada-Ogatta

**Affiliations:** ^1^Universidade Estadual de Londrina, Centro de Ciências Biológicas, Departamento de Microbiologia, Rodovia Celso Garcia Cid, km 380, 86057-970 Londrina, Brazil; ^2^Universidade Estadual de Maringá, Centro de Ciências da Saúde, Departamento de Ciências Básicas da Saúde, Maringá, Brazil

## Abstract

Most *Candida* spp. infections are associated with biofilm formation on host surfaces. Cells within these communities display a phenotype resistant to antimicrobials and host defenses, so biofilm-associated infections are difficult to treat, representing a source of reinfections. The present study evaluated the effect of eugenol on the adherence properties and biofilm formation capacity of *Candida dubliniensis* and *Candida tropicalis* isolated from the oral cavity of HIV-infected patients. All isolates were able to form biofilms on different substrate surfaces. Eugenol showed inhibitory activity against planktonic and sessile cells of *Candida* spp. No metabolic activity in biofilm was detected after 24 h of treatment. Scanning electron microscopy demonstrated that eugenol drastically reduced the number of sessile cells on denture material surfaces. Most *Candida* species showed hydrophobic behavior and a significant difference in cell surface hydrophobicity was observed after exposure of planktonic cells to eugenol for 1 h. Eugenol also caused a significant reduction in adhesion of most *Candida* spp. to HEp-2 cells and to polystyrene. These findings corroborate the effectiveness of eugenol against *Candida* species other than *C. albicans*, reinforcing its potential as an antifungal applied to limit both the growth of planktonic cells and biofilm formation on different surfaces.

## 1. Introduction

The prevalence of oral colonization by* Candida* spp. can vary among different population groups [1], and the presence of these fungi as commensals of human microbiota is one important predisposing factor for candidosis [2]. Adherence of the microorganisms to host cells and tissues is the first event required for initial colonization or establishment of infection. Moreover, the microbial surface contact can trigger various cellular behaviors, including biofilm formation [1], which is also strongly associated with candidosis [3].

Biofilms can be defined as irreversibly surface-attached communities of cells (sessile cells) embedded in a self-produced exopolymeric matrix, displaying a distinctive phenotype compared to their free-floating (planktonic cells) counterparts [4]. Remarkably, sessile cells are less susceptible to a variety of antifungal agents [5–7] and to host defenses [8]. Biofilms are thereby difficult to eradicate, representing a source of reinfections. Consequently, new antifungal agents are urgently needed, particularly those with antibiofilm activities, for effective management of* Candida* spp. infections.

Several researchers have shown the anti-*Candida* biofilm potential of plant-derived compounds such as flavonoids [9] and essential oils [10, 11]. Eugenol is the main active phenylpropanoid component of the essential oil from many aromatic plants [12]. The inhibitory effect of eugenol alone [13–17] and in combination with fluconazole and amphotericin B [18] against planktonic cells of* Candida* spp. has been previously reported. In addition, eugenol can interfere with the initial phases of biofilm formation, as well as with the viability of mature biofilm of* Candida albicans* [10, 11].

Although* C. albicans* continues to be the most common causative agent of candidosis in humans, other species of* Candida* have become a significant cause of such infections [19, 20].* Candida tropicalis* and* Candida dubliniensis* have been regarded as high biofilm producers, and sessile cells within this community have been found to be resistant to antifungal agents [5–7]. Accordingly, we analyzed the effect of eugenol on the hydrophobicity and adhesion to human epithelial cells and polystyrene of planktonic cells of these species. Moreover, the inhibitory activity against biofilm formation on polystyrene and denture materials was also analyzed.

## 2. Materials and Methods

### 2.1. *Candida* spp. Isolates and Growth Conditions

The* Candida* species used in this study included three* C. dubliniensis* (strains 131, 219, and 248) and three* C. tropicalis* (strains 23, 150, and 176) isolated from the oral cavity of HIV-infected patients. The species identification of oral isolates was carried out by standard mycological methods [21, 22]. Species identification was confirmed by a PCR-based method using specific primers as previously described [23, 24].* C. tropicalis* ATCC 28707 and* C. dubliniensis* ATCC MYA-646 type strains (kindly provided by FIOCRUZ, Rio de Janeiro, Brazil) were included as quality control. The isolates and strains were stored on Sabouraud dextrose (SD) agar and subcultured monthly. The yeasts were also maintained at −80°C. The study protocol was in accordance with the Ethics Committee of the Universidade Estadual de Londrina (CEP/UEL no. 036/10). Written informed consent was obtained from the patients for the publication of this report.

### 2.2. Biofilm Formation

*Candida *isolates were cultured in SD broth and incubated at 37°C for 18 h. The yeasts were harvested by centrifugation and washed twice with sterile 0.15 M phosphate-buffered saline, pH 7.2 (PBS), and the cells were counted in hemocytometric chamber (Neubauer Improved Chamber). A 20 *μ*L SD broth suspension of 6 × 10^5^ yeasts was placed into each well of flat-bottomed 96-well microtiter plates (Techno Plastic Products, Switzerland) containing 180 *μ*L of  SD broth. The plates were incubated at 37°C for 24 h. Afterwards, the wells were washed once with sterile distilled water, and the metabolic activity of the cells was quantified using the 2,3-bis(2-methoxy-4-nitro-5-sulfo-phenyl)-2H-tetrazolium-5-carboxanilide (XTT)-reduction assay. A 100 *μ*L aliquot of XTT-menadione (0.1 mg/mL XTT, 1 *μ*M menadione, Sigma Chemical Co, USA) was added to each well, and the plates were incubated in the dark for 2 h at 37°C. The XTT formazan product was measured at 490 nm with a microtiter plate reader (Universal Microplate Reader ELx 800, Bio-Tek Instruments, USA) [6]. To analyze the biofilm formation on denture material surfaces, the wells were aseptically coated with polymethylmethacrylate (PMM, OrtoClass, Clássico, Brazil) and ceramic (Noritake, Shofu Dental Corp., USA) before the assay.

### 2.3. Antifungal Susceptibility Testing

The growth inhibitory effect of eugenol (SSWhite, Brazil) on planktonic cells of* Candida *isolates was determined by broth microdilution assays according to the Clinical and Laboratory Standards Institute [25]. A stock solution of eugenol was prepared in water containing 10% dimethylsulfoxide (DMSO v/v, Sigma Chemical Co., USA). The DMSO final concentration in the assays did not exceed 1.0%. The substance was serially diluted 2-fold in RPMI buffered with MOPS, pH 7.0 (3000–5.85 *μ*g/mL eugenol). Quality control* C. dubliniensis* ATCC MYA-646 and* C. tropicalis* ATCC 28707 and fluconazole (512.0–0.5 *μ*g/mL, Pfizer Central Research, United Kingdom) were included in each experiment. Two wells of each plate served as growth and sterility controls. The minimum inhibitory concentrations (MICs) were determined at total inhibition of visual growth after 24 h incubation compared to untreated planktonic cells. To determine antifungal susceptibilities of sessile cells, biofilms were formed on polystyrene, as described above. After 1 and 24 h of biofilm formation, the medium was aspirated off and each well was washed three times with sterile PBS. A 200 *μ*L aliquot of RPMI 1640 medium containing serial 2-fold dilutions of eugenol and fluconazole was added, and the plates were further incubated for 24 h at 37°C. Controls included antifungal-free wells and biofilm-free wells. Sessile MICs were determined at 100% inhibition (SMIC_100_) compared to antifungal-free control wells using the XTT-reduction assay [6]. To evaluate the time-dependent effect of eugenol, 24 h biofilms of* Candida* species were formed in polystyrene and treated with SMIC_100_ of eugenol as described above. At determined time points (3, 6, 12, and 24 h), the metabolic activity of sessile cells was determined. All experiments were carried out in triplicate on three different occasions.

### 2.4. Cell Surface Hydrophobicity Determination

The hydrophobicity of untreated and eugenol-treated (0.5 × MIC for 1 h) planktonic cells was determined by the biphasic hydrocarbon/aqueous method according to Anil et al. [26]. Cell surface hydrophobicity (CSH) was expressed as the percentage decrease in optical density of the aqueous phase of the test as compared with the control, where the greater the change in absorbance of the aqueous phase, the more hydrophobic the yeast sample. Each assay was performed on three separate occasions with triplicate determinations each time.

### 2.5. Adhesion of Yeasts to HEp-2 Cells and Polystyrene

HEp-2 cells (human larynx epidermoid carcinoma) were cultured in Dulbecco's modified Eagle's medium (DMEM, Gibco) supplemented with 10% fetal bovine serum, 2 mM glutamine, 100 U/mL penicillin, 100 *μ*g/mL streptomycin, and 2.5 *μ*g/mL amphotericin B in a humidified 5% CO_2_ atmosphere at 37°C. For adhesion assays, HEp-2 cells were seeded in 24-well plates at 4.0 × 10^5^ cells per well and incubated for 18 h. The medium was removed and each well was washed three times with sterile PBS. The fresh culture medium minus the antimicrobials was added and the wells were inoculated with untreated and eugenol-treated* Candida *spp. (0.5 × MIC for 1 h) with approximately 2.0 × 10^6^ cells, and the plates were incubated at 37°C for 2 h in a 5% CO_2_ atmosphere. Nonadherent yeasts were removed by washing with sterile PBS. Adherent yeasts were harvested by treatment of the cell monolayers with 1 mL 0.5% (v/v) Triton X-100 (Sigma Chemical Co.) for 10 min on ice. The viable yeasts were enumerated by dilution plating in SD agar. Experiments were carried out in duplicate on three different occasions. The percent adherence was calculated by the equation: % Adherence = (cfu_120_/cfu_0_) × 100, where cfu_120_ refers to adhered cells per mL after 2 h and cfu_0_ the initial number of inoculated cells. The adhesion on polystyrene surface was performed as described for biofilm formation with minor modifications. Briefly, untreated and eugenol-treated (0.5 × MIC for 1 h) planktonic cells were placed in each well, and the plates were incubated for 2 h. The metabolic activity of adherent cells was determined using the XTT-reduction assay as described above.

### 2.6. Scanning Electron Microscopy (SEM)

Discs (0.8 cm diameter) of PMM and ceramic were aseptically placed in wells of 24-well tissue culture plates (Techno Plastic Products, Switzerland). A standard inoculum of 3.0 × 10^6^ cells, from overnight culture of the yeast, was prepared in 1 mL of RPMI 1640 medium, pH 7.0, and used to form biofilm on these surfaces. The discs were then immersed in these cell suspension and incubated statically at 37°C for 24 h. Afterwards, nonadherent organisms were removed by washing gently three times with PBS. One milliliter of RPMI containing eugenol (SMIC_100_) was added and the plates were further incubated for 24 h. Biofilms formed on these strips were fixed with 2.5% (v/v) glutaraldehyde in 0.1 M cacodylate buffer (pH 7.2) at room temperature. After fixation, the cells were dehydrated with a series of ethanol washes (15, 30, 50, 70, 80, 90, 95, and 100%), critical-point dried in CO_2_, coated with gold and examined with a SHIMADZU SS-550 scanning electron microscope.

### 2.7. Statistical Analysis

The results were evaluated by Student's* t*-test using the software GRAPHPAD PRISM version 5.0 (GRAPHPAD Software, San Diego, CA).* P* values less than 0.05 were considered significant.

## 3. Results

### 3.1. Biofilm Formation on Abiotic Surfaces

Biofilm formation by* Candida *species on polystyrene, PMM, and ceramic was monitored using the XTT-reduction assay. All isolates were able to form biofilms on these different substrate surfaces within 24 h, as assessed by the metabolic activity of sessile cells (Table 1). No significant differences in metabolic activities were observed between the strains and isolates in each substrate analyzed. However, a significant difference (*P* < 0.05) was observed between the substrates, where the highest biofilm formation was detected on polystyrene surface, followed by PMM and ceramic. The mean OD_490 nm_ (optical density at 490 nm ± standard deviation) was 1.032 ± 0.090 for the polystyrene, 0.777 ± 0.037 for PMM, and 0.598 ± 0.051 for ceramic surfaces.

### 3.2. Antifungal Activity against Planktonic and Sessile Cells

**Figure 1:**
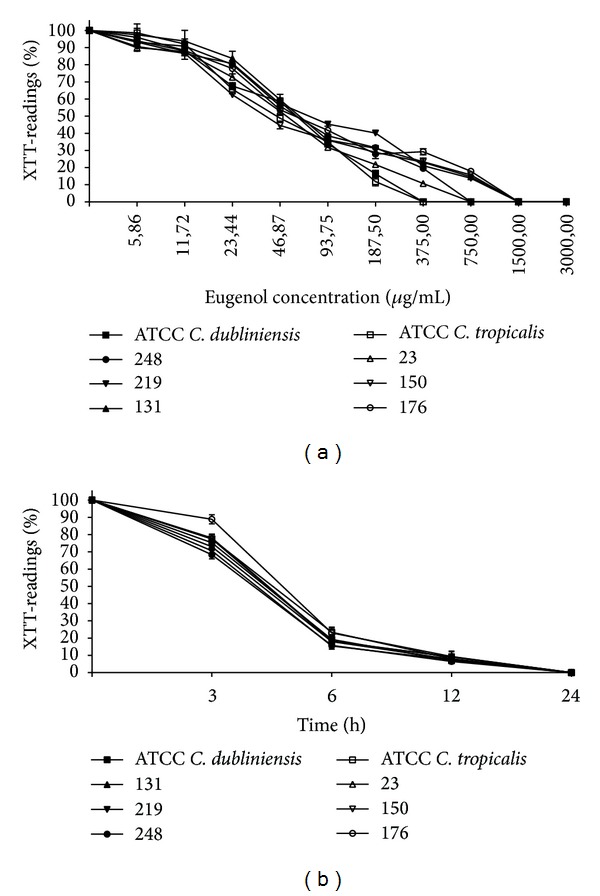
Effect of eugenol on viability of mature biofilm of* Candida dubliniensis *and* Candida tropicalis*. (a) The mature biofilms (24 h) were incubated in the presence of different concentrations (3000.0–5.86 *μ*g/mL) of eugenol for 24 h at 37°C. (b) The mature biofilms were incubated with SMIC_100_ concentrations of eugenol at 37°C and the metabolic activity of sessile cells was assessed at determined time points (3–24 h). Values are expressed as the average percentage of optical density (OD) of wells containing treated biofilms compared to that of control wells (considered to be 100%) for the XTT assays.

The MICs and SMICs of eugenol and fluconazole for the* Candida* spp. isolates and type strains are reported in Table 2. Planktonic cells of all isolates and the type strain of* C. dubliniensis* were susceptible to fluconazole. However, variation in fluconazole susceptibility was observed for* C. tropicalis*, where the reference type strain (ATCC 28707) and isolate 176 were resistant, isolate 150 dose-dependently susceptible, and isolate 23 susceptible to fluconazole, according to the CLSI [25] interpretative breakpoints. The biofilm of these* Candida* species exhibited high resistance to fluconazole. The SMIC_100_ of this compound for all isolates and type strains was higher than 512 *μ*g/mL. The MIC values of eugenol for* C. dubliniensis* and* C. tropicalis* planktonic cells ranged from 375 to 750 *μ*g/mL. Trailing growth was observed when fluconazole was tested against all* C. tropicalis* strains, while eugenol completely inhibited the growth of planktonic cells. Eugenol also exhibited an inhibitory effect against mature biofilms of* Candida* species, which appeared to be dose dependent (Figure 1(a)). There was a more than 80% reduction in metabolic activity of 24 h sessile cells with eugenol at concentrations of 187.5 to 750 *μ*g/mL. No metabolic activity was detected at concentrations ranging from 375 to 1500 *μ*g/mL, and these values were considered the SMIC_100_. The inhibitory effect of eugenol against 24 h sessile cells was also time dependent (Figure 1(b)). The reduction in metabolic activity ranged from 11.1 to 31.6%, 76.6 to 85.5%, and 90.6 to 93.5% after incubation in the presence of SMIC_100_ eugenol for 3, 6, and 12 h, respectively. No detectable metabolic activity was observed after 24 h treatment. Eugenol also interfered with biofilm formation, since treatment of 1 h adherent cells resulted in dose-dependent reduction of their metabolic activity (data not shown). The SMIC_100_ for 1 h adherent cells ranged from 375 to 750 *μ*g/mL (Table 2).

### 3.3. Scanning Electron Microscopy of* Candida tropicalis* Biofilm on Denture Materials

**Figure 2:**
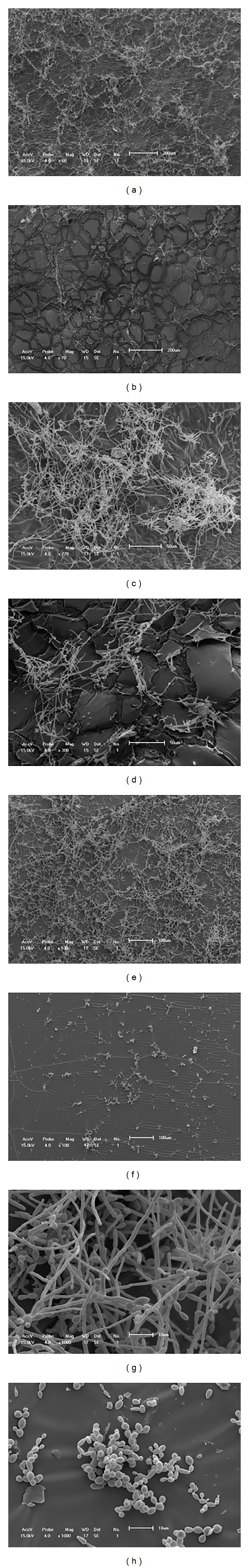
Scanning electron microscopy images of the effect of eugenol on* Candida tropicalis *mature biofilm formed on the surface of polymethylmethacrylate ((a)–(d)) and ceramic ((e)–(h)). Untreated mature biofilms ((a), (c), (e), and (g)) and treated biofilms with eugenol-SMIC_100_ for 24 h ((b), (d), (f), and (h)).

The effect of eugenol on* C. tropicalis *(isolate 150, MIC of fluconazole = 32 *μ*g/mL) biofilms formed on PMM and ceramic surfaces was monitored by SEM (Figure 2). Mature biofilms of untreated cells of this isolate consisted of a dense network of cells, composed of a heterogeneous layer of yeast, pseudohyphae, and hyphae (Figures 2(a), 2(c), 2(e), and 2(g)). The treatment of biofilms with eugenol drastically reduced the amount of sessile cells of* C. tropicalis* on the denture materials surfaces (Figures 2(b), 2(d), 2(f), and 2(h)).

### 3.4. Effect of Eugenol on Cell Surface Hydrophobicity and Adhesion to HEp-2 Cells and Polystyrene

To evaluate the effect of eugenol on CSH and adhesion to mammalian cells and polystyrene, planktonic cells of* Candida* species were exposed to eugenol at a subinhibitory (0.5 × MIC) concentration for 1 h before the assays. Most* Candida* spp. isolates showed hydrophobic behavior as determined by the biphasic hydrocarbon/aqueous method, and the mean relative CSH was 60.82 ± 20.79 ranging from 29.48 ± 2.97 to 84.59 ± 4.32. Except for* C.  tropicalis* 176, a significant difference (*P* < 0.005) in CSH of* Candida *species was observed after exposure of planktonic cells to eugenol for 1 h (Table 3). There was a range of 42.3 to 75.1% reduction in the CSH of eugenol-treated cells as compared to untreated counterpart cells. Eugenol also caused a significant reduction in adhesion of most* Candida* species to HEp-2 cells (*P* < 0.005) and to polystyrene (*P* < 0.05). There was no significant difference in the adhesion percentage of isolate 176 of* C. tropicalis *to either surface, although a 20% reduction in adhered cells to mammalian cells was seen after eugenol exposure. The other* Candida* isolates showed a range of 46.9 to 68.9% and 27.4 to 67.8% reduction in adhesion to HEp-2 cells and polystyrene, respectively.

## 4. Discussion

Eugenol has been widely used in medicine and dentistry due to its antiseptic, antimicrobial, anesthetic, analgesic, antioxidant, anti-inflammatory, and cardiovascular properties [27, 28]. This phenylpropanoid compound has been reported to have antimicrobial activity against planktonic cells of* C. albicans, C. dubliniensis, C. glabrata*,* C. guilliermondii*,* C. krusei*,* C. parapsilosis, *and* C. tropicalis* [13–17, 29]. Moreover, this compound shows* in vitro* synergy with fluconazole and amphotericin B against* C. albicans* [11, 18]. As previously reported [14–17], our results showed that eugenol has fungicidal activity against planktonic cells of* C. tropicalis*, including those classified as fluconazole-resistant and dose-dependent yeasts, and this effect was also observed for* C. dubliniensis*.

Previous studies reported in the literature have focused on determining the antibiofilm activity of eugenol against* C. albicans*. He et al. [10] showed a dose-dependent reduction in metabolic activity of 48 h biofilm formed on a polystyrene surface and treated with eugenol for another 48 h. In the presence of 500 *μ*g/mL and 2000 *μ*g/mL eugenol, 50% (SMIC_50_) and over 80% (SMIC_80_) reduction were detected, respectively. Khan and Ahmad [11] evaluated the effect of phytocompounds (eugenol, cinnamaldehyde, citral, and geraniol) against 48 h biofilm of* C. albicans*, and their results also showed a dose- and time-dependent inhibitory activity for eugenol. The SMIC_80_ after treatment with the compounds for 48 h ranged from 100 to 400 *μ*g/mL. The results obtained in this study showed that eugenol displayed inhibitory activity against biofilms of* C. dubliniensis *and* C. tropicalis*, which, not surprisingly, were highly resistant to fluconazole. Eugenol inhibited biofilm formation, as well as reducing metabolic activity of mature biofilms formed on polystyrene, in a dose-dependent manner. SEM analysis further revealed the reduction in biofilm formed on denture materials (PMM and ceramic).

The mechanisms by which eugenol induces death in* Candida* spp. are not completely understood. This compound caused profound changes in the morphology of planktonic cells and leakage of cytoplasmic constituents, indicating an action on the cell envelope [13, 14]. In fact, several authors have shown that the fungicidal concentration of eugenol against* C. albicans *causes a significant reduction in ergosterol content of the cell [15, 16, 30] and interferes with H^+^-ATPase activity [31]. In addition, the extensive damage to the cell membrane [15, 30] may be attributed to oxidative stress mediated by reactive oxygen species [29].

Microbial adherence on the surface of substrates is the initial event of biofilm formation, and the cell envelope mediates the first interaction between the microorganism and the environment. CSH, a nonspecific factor, is considered an important feature that contributes to adherence of* Candida* spp. on different surfaces [32, 33]. Moreover, it has been shown that CSH of planktonic cells of* C. albicans* isolated from different sources correlates positively with biofilm formation on polystyrene [34]. In this study, the presence of eugenol (0.5 × MIC) caused a significant reduction in CSH and adhesion to polystyrene and HEp-2 cells of almost all planktonic cells of* C. tropicalis* and* C. dubliniensis*. These results suggest that eugenol may interfere with the adhesion properties of* Candida* species. It was previously reported that* C. albicans* adhesion to polystyrene [35] and epithelial cells [36] was reduced after* in vitro* exposure to subinhibitory concentrations of fluconazole, an antifungal that interferes with ergosterol biosynthesis.

Altogether, the findings reported here corroborate the effectiveness of eugenol against planktonic and sessile cells of* Candida* species other than* C. albicans,* reinforcing the potential of this compound as an antifungal, indicating that this phenylpropanoid may have additional beneficial effect in the treatment of local candidiasis. Accordingly, initial* in vivo* studies have demonstrated the safety and efficacy of the topical use of eugenol for the treatment of vaginal [37] and oral [14] candidosis in rats. Further studies are warranted to confirm its efficacy in the prophylaxis and/or treatment of biofilm-associated candidosis in human.

## 5. Conclusion

The results obtained in this study showed that besides having fungicidal activity, eugenol is capable of changing the CSH and adhesion capacity of planktonic cells of* C. dubliniensis *and* C. tropicalis*. In addition, this phenylpropanoid compound inhibited biofilm formation and mature biofilm formed on polystyrene and denture materials of both* Candida* species.

## Figures and Tables

**Table 1:** Metabolic activities of biofilm formed by *Candida dubliniensis* and *Candida tropicalis* on different substrate surfaces.

Isolate	Metabolic activity (OD)^a^		
	Polystyrene	PMM	Ceramic
*Candida dubliniensis *			
ATCC MYA-646	0.855 ± 0.029	0.711 ± 0.056	0.499 ± 0.055
131	1.045 ± 0.032	0.795 ± 0.058	0.628 ± 0.056
219	0.989 ± 0.033	0.751 ± 0.054	0.566 ± 0.057
248	1.094 ± 0.034	0.810 ± 0.055	0.637 ± 0.056
*Candida tropicalis *			
ATCC 28707	0.978 ± 0.029	0.745 ± 0.058	0.559 ± 0.056
23	1.136 ± 0.032	0.815 ± 0.056	0.638 ± 0.057
150	1.100 ± 0.034	0.801 ± 0.056	0.635 ± 0.057
176	1.056 ± 0.031	0.786 ± 0.057	0.624 ± 0.054

Mean ± SD	1.032 ± 0.090*	0.777 ± 0.037^#^	0.598 ± 0.051^**¥**^

^a^Metabolic activity of sessile cells was determined by the XTT-reduction assay. The XTT formazan product was measured at 490 nm.

^∗,#,**¥**^Means not sharing a symbol differ significantly (*P* < 0.05) between the abiotic surfaces.

**Table 2:** Antifungal concentrations of eugenol and fluconazole against planktonic and sessile cells of *Candida dubliniensis* and *Candida tropicalis. *

Yeast	Eugenol			Fluconazole	
	MIC^a^	SMIC-1^b^	SMIC-24^c^	MIC^d^	SMIC^c^
*Candida dubliniensis *					
ATCC MYA-646	375	375	375	8	>512
131	750	750	1,500	4	>512
219	375	750	1,500	8	>512
248	375	750	750	4	>512
*Candida tropicalis *					
ATCC 28707	375	375	375	128	>512
23	375	750	750	8	>512
150	750	750	1,500	32	>512
176	375	750	1,500	64	>512

^a^Minimum inhibitory concentration of the antifungal which resulted in total inhibition of visible planktonic cell growth; ^b^Minimum inhibitory concentration of the antifungal which resulted in total reduction in metabolic activity of sessile cells, using the XTT-reduction assay, after 1 h of adhesion; ^c^Minimum inhibitory concentration of the antifungal which resulted in total reduction in metabolic activity of sessile cells, using the XTT-reduction assay, after 24 h of biofilm formation; ^d^MIC was defined according to CLSI (2008) guidelines for fluconazole broth microdilution assays; The results are expressed as *μ*g/mL.

**Table 3:** Effect of eugenol on cell surface hydrophobicity, and adhesion to human epithelial cells and polystyrene.

Isolate	CSH^a^		Adhesion to HEp-2 cells^b^		Adhesion to polystyrene^c^	
	Untreated	Treated^d^	Untreated	Treated^d^	Untreated	Treated^d^
*Candida dubliniensis *						
131	67.97 ± 5.61*	39.22 ± 6.97	92.00 ± 5.60^#^	35.00 ± 5.27	0.450 ± 0.001′′	0.302 ± 0.001
219	29.48 ± 2.97*	15.58 ± 3.16	45.00 ± 4.16^#^	14.00 ± 4.53	0.405 ± 0.002′′	0.209 ± 0.001
248	69.20 ± 9.10*	16.00 ± 6.11	90.00 ± 5.21^#^	30.00 ± 4.73	0.384 ± 0.001′′	0.216 ± 0.002
*Candida tropicalis *						
23	72.00 ± 8.22*	21.00 ± 5.63	92.00 ± 5.12^#^	46.00 ± 4.33	0.397 ± 0.004′′	0.288 ± 0.003
150	41.66 ± 4.72*	23.75 ± 5.21	81.00 ± 5.06^#^	43.00 ± 5.84	0.335 ± 0.002′′	0.108 ± 0.001
176	84.59 ± 4.32	81.16 ± 3.19	45.00 ± 3.12	36.00 ± 3.21	0.395 ± 0.005	0.393 ± 0.002

^a^Percentage of cell surface hydrophobicity (CSH) determined by the difference in the optical density (OD) of the aqueous phase between test and control. The greater the change in OD of the aqueous phase, the more hydrophobic the yeast sample is. ^b^The percent adherence was calculated by the equation: % Adherence = (cfu_120_/cfu_0_) × 100, where cfu_120_ refers to adhered bacterial cells per mL after 2 h and cfu_0_ the initial number of inoculated cells. ^c^The metabolic activity of cells was determined by the XTT-reduction assay after 2 h of adhesion on polystyrene surface. ^d^Planktonic cells were eugenol-treated for 1 h with 0.5 × MIC before the assay. Significant differences in CSH (∗), adhesion to HEp-2 cells (#) and to polystyrene (′′) properties when compared to eugenol-treated counterpart cells (^∗,#^ *P* < 0.005; ′′*P* < 0.05).
